# Establishing the prevalence of low vitamin D in non-immunoglobulin-E mediated gastrointestinal food allergic children in a tertiary centre

**DOI:** 10.1186/s40413-016-0135-y

**Published:** 2017-01-11

**Authors:** Ru-Xin Foong, Rosan Meyer, Robert Dziubak, Adriana Chebar Lozinsky, Heather Godwin, Kate Reeve, Syeda Tahmida Hussain, Romman Nourzaie, Neil Shah

**Affiliations:** 1 Paediatric Gastroenterology Department, Great Ormond Street Hospital for Children NHS Foundation Trust, London, UK; 2 Institute of Child Health, University College, London, UK; 3 Imperial College of London, London, UK; 4 Barts and the London School of Medicine and Dentistry, Queen Mary University of London, London, UK

**Keywords:** Non-IgE mediated allergy, Vitamin D, Paediatrics

## Abstract

**Background:**

There is no data on the prevalence of vitamin D deficiency in children with non-immunoglobulin-E (IgE) mediated gastrointestinal food allergy. The aims of our study were to understand the prevalence of vitamin D insufficiency and deficiency in children with non-IgE mediated gastrointestinal food allergy and identify predisposing factors.

**Methods:**

This was a retrospective study which looked at data from Great Ormond Street Hospital from January 2002 to September 2015. Children 0–18 years old with a confirmed diagnosis of non-IgE mediated gastrointestinal food allergy who had a vitamin D level measured during the course of their disease were included. Low vitamin D levels were defined as <50 nmol/L; insufficient levels were defined as 25–50 nmol/L and deficient levels as <25 nmol/L. Patient characteristics and clinical factors were also recorded.

**Results:**

Ninety-two patients met the study criteria; 49% were female and median age was 10 years 2 months [IQR: 4 years 8 months to 13 years 7 months]. Of the cohort, 26% (24/92) had low vitamin D levels; 16% had insufficient vitamin D levels and 10% had vitamin D deficiency. Gender (*p* = 0.043) and age (*p* = 0.035) were significantly associated with low vitamin D levels. Twelve percent of children who were on an amino acid formula (AAF) had low vitamin D compared to 31% of children who were not (*p* = 0.06). No other clinical factors were found to be significantly associated with low vitamin D levels.

**Conclusions:**

Children with non-IgE mediated gastrointestinal food allergy are at risk of vitamin D insufficiency and deficiency. Further prospective studies need to be performed in all children with non-IgE mediated gastrointestinal food allergies.

**Trial registration:**

The study was registered with the GOSH Research & Development department as a retrospective case note review. The Health Research Authority confirmed that NHS Research and Ethics Committee approval was not required; thus there is no trial registration number.

## Background

Vitamin D is a fat-soluble vitamin acquired from exposure to sunlight through skin synthesis and diet [1, 2]. The role of vitamin D within the immune system has been well described and its association with food allergy has been of great interest in recent years [1, 3]. Vitamin D is thought to improve antimicrobial defences within the immune system, suppress excessive inflammation and inhibit production of pro-inflammatory cytokines. More specifically, it is thought to help maintain epithelial barrier integrity by regulating tight junction proteins. It has been hypothesised that children with vitamin D deficiency are more likely to have dysbiotic microbial flora, increased risk of gastrointestinal infections and an abnormal intestinal barrier increasing the risk of developing food allergy [4].

Various factors can affect vitamin D levels in children, including dietary intake, ethnicity, sun exposure, being born in the winter and obesity [5]. Dietary intake can lead to low levels through extended breastfeeding without supplementation, late introduction of hen’s egg or a low maternal consumption of oily fish in pregnancy. Varying levels of vitamin D fortification of food and infant supplementation may also impact on vitamin D levels [2, 5].

Immunoglobulin E (IgE)-mediated food allergy tends to have a quick onset presenting with skin, respiratory and gastrointestinal symptoms whereas non-IgE mediated food allergy has a delayed onset of symptoms that are usually skin and gastrointestinal in nature [6]. Previous studies have shown an association between children with vitamin D deficiency and increased risk of IgE-mediated food allergy sensitisation, [4, 7, 8] conversely children with food allergy were at greater risk of vitamin D deficiency [9, 10]. An Australian study established that children with vitamin D insufficiency (<50 nmol/L) were more likely to be sensitised to both egg and peanut, but also at a greater risk of having multiple food allergies [11]. However, the majority of these studies have focused on the role of vitamin D in association with IgE-mediated food allergy with limited studies specifically focusing on the relationship of vitamin D and non-IgE mediated gastrointestinal food allergy. Similar to IgE-mediated food allergy, dietary elimination forms the mainstay of management in non-IgE-mediated food allergy, which can lead to restricted intake [12, 13]. Meyer et al. [13] found that in non-IgE gastrointestinal food allergic children on an elimination diet, there was a high risk of insufficient vitamin D intake especially in children not on a hypoallergenic formula [14]. It may also be that children with non-IgE gastrointestinal food allergy are at risk of developing vitamin D deficiency given the changes that occur to the gut on exposure to food antigens which can impact the role vitamin D metabolites have on the epithelial defences of the gut [4, 15]. In a study by Slack et al. [8], vitamin D levels were found to be low in patients with eosinophilic oesophagitis, especially those that were older and had a higher body mass index.

The main aim for this study was to understand the prevalence of vitamin D insufficiency and deficiency in children with non-IgE mediated gastrointestinal food allergy. We also wanted to identify predisposing factors for vitamin D deficiency in these children.

## Methods

A retrospective study was performed at Great Ormond Street Hospital (GOSH), United Kingdom, from January 2002 to September 2015. The study was registered and approved by GOSH Research and Development department. All children aged 0–18 years old with a diagnosis of non-IgE mediated gastrointestinal food allergy established through an elimination diet [16] of at least one of the common allergic foods (i.e. cow’s milk, soya, egg, wheat, gluten, nuts, fish) were identified from the gastroenterology department’s clinical database of patients. Of this group, those who had a vitamin D level performed at any point during the course of their disease were included. Patients with concomitant non-atopic co-morbidities that could influence vitamin D intake or mechanism (chronic kidney or liver disease, history of prematurity, comorbidities affecting food intake (i.e cerebral palsy, history of bowel surgery) were excluded. Included patients had their demographics, clinical details and blood results entered into an anonymized, secure electronic database. This included data on gastrointestinal symptoms (diarrhoea, rectal bleeding, reflux/vomiting, constipation, abdominal pain, flatus, bloating/distension, back arching, food aversion), food elimination diets and/or hypoallergenic formulas and any evidence of histology results from endoscopy. Blood tests recorded included vitamin D levels, Parathyroid Hormone, Alkaline phosphatase, Ferritin, Haemoglobin, vitamins A, E and B12, Folate, Zinc, Selenium and Copper. If multiple micronutrient blood results were available, the test closest to the date the vitamin D sample was taken was recorded. Weight and height/length measurements were recorded at the time their vitamin D level was captured. Z-scores were calculated using the World Health Organisation (WHO) Anthro (Version 3.2.2) or AnthroPlus (Version 1.0.4) software according to age. We used the WHO guidelines to define undernutrition at ≤ -2- z-score and overnutrition ≥ 2 z-score [17]. Low vitamin D was defined as <50 nmol/L, more specifically vitamin D insufficiency was defined as 25–50 nmol/L and vitamin D deficiency as <25 nmol/L [11].

### Statistical analysis

Statistical analysis was performed using IBM SPSS Statistics for Windows, Version 22 (Armonk, NY). Continuous variables are presented as medians with interquartile ranges (IQR) or means where appropriate and categorical variables are presented as percentages. Mann Whitney U-tests were used to compare age and total number of dietary foods eliminated between the two groups. Pearson’s chi square test or Fisher’s Exact test were used where appropriate to compare gastrointestinal symptom presentation, atopic comorbidities, the type of dietary foods eliminated, the type of hypoallergenic formulas used, ethnicity and other vitamin and mineral bloods between children with normal vitamin D levels and those with insufficient levels. All tests were two-sided and significance level was set to 0.05.

## Results

**Fig. 1:**
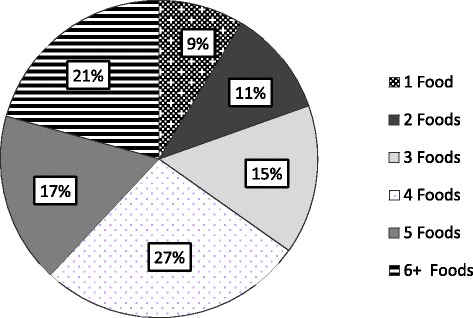
The percentage of children who had specific numbers of foods eliminated

The medical records of 711 children that were within the inclusion criteria were reviewed; 114 children were excluded due to concomitant non-atopic co-morbidities as described in the methodology. Of the 505 children remaining, 92 (18%) had a vitamin D level measured. Of this group 49% (45/92) were female and the median age was 10 years 2 months [IQR: 4 years 8 months to 13 years 7 months] at the time their vitamin D level was measured. Within the group, 71% of the children were Caucasian, 13% Asian, 4% Black Afro-Caribbean and 12% did not specify their ethnicity. Information regarding foods eliminated, formulas the children were on, gastrointestinal symptoms experienced and atopic co-morbidities is shown in Table 1. The number of foods eliminated for the cohort is shown in Fig. 1. The median weight-for-age z-score for the children who had anthropometric data available at the time their vitamin D level was measured (*n* = 51/92, 55%) was: -0.82 [IQR: -1.64 to 0.79] and median height-for-age z-score was -1.1 [IQR: -1.93 to 0.26] (*n* = 60/92, 65%). Ten children (20%) had weight-for-age z-scores lower than -2 and 14 children (23%) had height-for-age z-scores lower than -2. There were no significant differences in z-scores between children with normal or low vitamin D levels, *p* = 0.905 and *p* = 0.422 respectively.

**Table 1:** Descriptive statistics of our cohort

Variable	Number of patients (%)
Type of food eliminated	
Cow’s milk	86 (94)
Gluten (including wheat)	71 (77)
Egg	65 (71)
Soya	64 (70)
Nuts	10 (11)
Fish	5 (5)
Type of formula	
Amino acid formulas	25 (26)
Over-the-counter milks	21 (22)
Extensively hydrolyzed (whey/casein) formulas	8 (8)
Breast milk only	2 (2)
GI symptoms	
Abdominal pain	63 (69)
Vomiting	56 (61)
Diarrhoea	48 (52)
Constipation	29 (32)
Faltering growth	24 (26)
Bloating/abdominal distension	16 (17)
PR Bleeding	11 (12)
Food aversion	11 (12)
Flatus	10 (11)
Back arching	3 (3)
Atopic co-morbidities	
Eczema/atopic dermatitis	38 (45)
Asthma	20 (24)
Allergic rhinitis	16 (19)

Of the cohort, 26% (24/92) had low vitamin D levels as per our criteria (<50 nmol/L) but more specifically, 16% (15/92) had insufficient vitamin D levels (25–50 nmol/L) and 10% (9/92) were vitamin D deficient (<25 nmol/L).

Analysis of the data indicated that age was significantly associated with low vitamin D levels (*p* = 0.035). More specifically, we found that children with low vitamin D were older (median age 12 years 5 months [IQR: 9 years 2 months to 14 years 4 months) than children with normal vitamin D levels (median age: 8 years 5 months [IQR: 4 years 4 months to 12 years 9 months]. Being female was also associated with low vitamin D (*p* = 0.043). However, we did not find any statistical significance between low vitamin D levels and ethnicity or the type and number of foods eliminated. Children on an amino acid formula (AAF) were less likely to have a low vitamin D level compared to children not on an AAF, 31% versus 12% respectively (*p* = 0.06). The median age of the children on an AAF was 8.1 years compared to 10.7 years for those not on an AAF. The majority of the type of gastrointestinal symptoms at presentation, atopic co-morbidities and abnormal histology from biopsy results were also found not to be associated with low vitamin D. However, significantly more children who did not have vomiting as a presenting gastrointestinal symptom (42%) had a low vitamin D level compared to those who had vomiting symptoms (16%) (*p* = 0.006).

We also collected data on other nutritional bloods from these patients. Of those who had nutritional bloods available, 71% had high folate levels, but up to 19% had vitamin A, selenium, copper and zinc deficiencies (Table 2). There was no significant association found between low vitamin D and having any abnormal nutritional bloods (*p* > 0.232).

**Table 2:** Percentage of normal and abnormal nutritional bloods for entire cohort

Blood test	Number	Normal	Abnormal	Abnormal (Low)	Abnormal (High)
Vitamins					
Vitamin A	43	81%	19%	19%	0%
Vitamin E	41	93%	7%	2%	5%
Vitamin B12	29	69%	31%	3%	28%
Folate	7	29%	71%	0%	71%
Minerals					
Zinc	48	83%	17%	15%	2%
Copper	45	78%	22%	16%	7%
Selenium	43	81%	19%	19%	0%
Other nutritional bloods					
Haemoglobin	89	82%	18%	15%	3%
Ferritin	69	84%	16%	7%	9%
Alkaline phosphate	41	61%	39%	20%	20%
Parathyroid Hormone	14	79%	21%	7%	14%

## Discussion

There is evidence suggesting that food allergic children are at risk of developing vitamin D deficiency but there is paucity of data in children with non-IgE mediated gastrointestinal food allergies [9, 10]. To our knowledge this is the first study that has specifically looked at vitamin D levels in a cohort of non-IgE mediated gastrointestinal food allergic children. In this retrospective study, we found that over 26% of our cohort had low vitamin D levels and 10% of the 26% were vitamin D deficient.

Two factors were significantly associated with vitamin D insufficiency: age and gender. It is already known that females, particularly teenagers and young women [2, 18] are at a higher risk of developing this deficiency and our data suggest that young girls with food allergy are also at a higher risk, although more boys have food allergy (64%) [19]. Our data indicates that girls are at greater risk of low vitamin D in gastrointestinal food allergy.

Although children less than 5 years old in the general population are at higher risk for vitamin D deficiency [2, 20], we found that in our allergic cohort older children were at higher risk. Older children who have non-IgE mediated gastrointestinal food allergy tend to be the children with more persistent and complex allergic disease. They often have more investigations performed and may be on elimination diets for longer. Some of the common foods eliminated (i.e. milk, egg) are high in vitamin D and if not adequately substituted can increase the risk of low vitamin D levels. Slack et al. [8] investigated vitamin D levels in adult and paediatric patients with eosinophilic oesophagitis and found that in their cohort, patients with insufficient vitamin D levels were older compared to those with sufficient levels, median age 25.5 and 16.2 years respectively. The UK recommends multivitamins with vitamin D for children below the age of 5 years, providing these for free for those with insufficient financial means [21]; however, for older children there is no such recommendation and no free vitamin supplementation scheme which may also explain the greater risk of deficiency. In a US-based study performed on food allergic children, 25% of children consumed less than 67% of the Recommended Dietary Allowance of vitamin D and calcium and they were also found to be shorter [22]. Although not statistically significant, 23% of our entire cohort had z-scores lower than -2. Studies have shown that food allergic children have a greater risk of impaired growth [12, 23, 24], but the fact that this may also be associated with poor growth in the non-IgE mediated cohort requires further attention.

We also found that a lower percentage of the children on an AAF had vitamin D deficiency compared to those children who were on alternative feeds (12% versus 31%). The median age of the children in the whole cohort on AAF was 8.1 years which is relatively high to still be on an AAF, likely indicating the severity and persistence of their disease. In addition vitamin D levels in hypoallergenic formulas and over the counter milks vary greatly, which may explain our findings, preferring an AAF for vitamin D. The bioavailability and source of vitamin D may also play a role. Although the UK does not routinely fortify foods with vitamin D, it could be that the fortification of hypoallergenic foods suitable for older children and routine vitamin D supplementation for children with non-IgE mediated food allergy could be beneficial.

There were no other nutritional blood tests that were significantly associated with low vitamin D levels. However, up to 19% had micronutrient deficiencies and many of these children were on multiple food elimination diets. Previous studies looking at food allergy have shown these children to be at risk of nutritional deficiencies, with low levels of vitamin A, selenium and zinc [13, 25], which have also been seen in children with other atopic conditions such as eczema [26]. These micronutrients have important immunomodulatory effects in the body and should be monitored in children with gastrointestinal food allergy [13, 25].

### Limitations

One of the key limitations in this study was that it was retrospective and our data relied on the quality of the clinical notes available. The timing at which the vitamin D level was measured varied from patient to patient and was clinician dependant, which could have biased the results. There are also other variables that could have affected vitamin D levels such as sun exposure, seasonal timing of when the test was performed, other dietary factors which would be useful to consider in future studies in this population of children. Our cohort was also relatively small with a total of 92 patients included, which may have been why we were unable to identify any factors that had a significant impact on vitamin D levels. However, the fact that 10% of the children were vitamin D deficient is a novel finding and needs addressing in the future. There is also no current policy for vitamin D testing; in our study, the decision to measure vitamin D was based on suspicion of nutritional deficiency by the clinician reviewing the child. There is the possibility that children who are deficient are being missed further emphasising the importance of more research into this cohort of patients.

## Conclusion

Children with non-IgE mediated gastrointestinal food allergy seem to be at risk of low vitamin D levels especially older, female patients. It is worth checking the levels in these children with persistent allergy, irrespective of the number of foods eliminated, but especially if they are not on an an AAF. Further prospective research needs to be performed in all children with non-IgE mediated gastrointestinal food allergies, not only children deemed at risk by clinical judgement.

## Abbreviations

AAF: Amino acid formula
GOSH: Great Ormond Street Hospital
IgE: Immunoglobulin-E
WHO: World Health Organisation
